# The limits of market-based reforms in the NHS: the case of alternative providers in primary care

**DOI:** 10.1186/1472-6963-13-S1-S3

**Published:** 2013-05-24

**Authors:** Anna Coleman, Kath Checkland, Imelda McDermott, Stephen Harrison

**Affiliations:** 1 Health Policy, Politics & Organisation research group (HiPPO), Centre for Primary Care, Institute of Population Health, University of Manchester, 5th Floor Williamson Building, Oxford Road, Manchester, M13 9PL, UK

## Abstract

**Background:**

Historically, primary medical care in the UK has been delivered by general practitioners who are independent contractors, operating under a contract, which until 2004 was subject to little performance management. In keeping with the wider political impetus to introduce markets and competition into the NHS, reforms were introduced to allow new providers to bid for contracts to provide primary care services in England. These contracts known as ‘Alternative Provider Medical Services’, were encouraged by two centrally-driven rounds of procurement (2007/8 and 2008/9). This research investigated the commissioning and operation of such Alternative Providers of Primary Care (APPCs).

**Methods:**

Two qualitative case studies were undertaken in purposively sampled English Primary Care Trusts (PCTs) and their associated APPCs over 14 months (2009-10). We observed 65 hours of meetings, conducted 23 interviews with PCT and practice staff, and gathered relevant associated documentation.

**Results and conclusions:**

We found that the procurement and contracting process was costly and time-consuming. Extensive local consultation was undertaken, and there was considerable opposition in some areas. Many APPCs struggled to build up their patient list sizes, whilst over-performing on walk-in contracts. Contracting for APPCs was ‘transactional’, in marked contrast to the ‘relational’ contracting usually found in the NHS, with APPCs subject to tight performance management. These complicated and costly processes contrast to those experienced by traditionally owned GP partnerships. However, managers reported that the perception of competition had led existing practices to improve their services.

The Coalition Government elected in 2010 is committed to ‘Any Qualified Provider’ of secondary care, and some commentators argue that this should also be applied to primary care. Our research suggests that, if this is to happen, a debate is needed about the operation of a market in primary care provision, including the trade-offs between transparent processes, fair procurement, performance assurance and cost.

## Background

Since 1948, National Health Service (NHS) Primary Medical Services in England have been provided by General Practitioners (GPs), via the General Medical Services (GMS) contract. This contract is centrally negotiated and flexible in its operation, with little specification of the details of services to be provided. Primary medical services (PMS) contractors are required to provide patients with essential services, which are loosely defined, and most also provide a menu of additional and enhanced services which are paid for in addition to the core contract. GP practices act as both the gateway to and co-ordinator of patient access throughout their care journey. They are usually the first point of contact for a patient seeking treatment or advice about their health, and most work together in practices owned by one or more self-employed GPs and which employ additional clinical staff such as nurses and healthcare assistants. Payment for the contract is worked out from a formula based on the practice population profile, taking into account factors such as age and gender of patients and list turnover (number of registered patients) of each practice, rather than the number of doctors.

A series of reforms in the NHS since 1991 have encouraged market-like behaviour to develop, splitting purchasers (commissioners) and providers of health services [1]. As part of these reforms, Alternative Provider Medical Services (APMS) contracts were introduced in 2004, permitting primary care to be commissioned from organisations other than traditional general medical practices (though the new form of contract may also be held by the latter). The initial logic underlying this move was to enhance the provision of services for groups underserved by traditional general practices, such as the homeless [2]. APMS contracts differed from the centrally-negotiated GMS contracts in that Primary Care Trusts (PCTs) were able to negotiate the exact terms of the contract locally.

Initially, few APMS practices were procured, but in 2007 the Government acted to stimulate entry of new providers into the primary care market by introducing the ‘Fairness in Primary Care Procurement’ (FPCP) process into PCT areas deemed to be ‘under-doctored’. This was followed in 2008 by the ‘Equitable Access to Primary Medical Care’ (EAPMC) initiative. Ten PCTs participated in FPCP, with most of these procuring a single new practice [3]. Under EAPMC, 112 new practices in 50 PCTs were procured. Successful bidders for these contracts included private companies, social enterprises and other mutual organisations, groups of existing GPs, and organisations integrating with other NHS providers such as Foundation Trusts and providers of out-of-hours care. Under these two rounds of procurement, a standard APMS contract template was developed by the Department of Health, although elements of this could still be subject to local negotiation [4].

Officially, FPCP was developed to deliver the commitment in the 2006 White Paper *Our Health*, *Our Care*, *Our Say *[2] to tackle inequalities in access to primary medical care services in the most under-doctored PCTs throughout England. This round of procurement was expected to provide patients with greater access and choice, including flexible opening hours, extended services and easier access to primary medical care services in their local area [5]. EAPMC arose from the interim report of the NHS Next Stage Review (NSR) [6], committing the NHS to establish at least 150 ‘GP-led Health Centres’ (one in each PCT area) which would provide access to GP services, including walk-in services and pre-bookable GP appointments, from 8am to 8pm, seven days a week. The NSR also stated that, in addition to the above, the NHS would establish at least 100 new GP practices in the 25 percent of the country with the poorest primary care provision.

This paper reports a qualitative study of the commissioning and subsequent monitoring of primary care from organisations other than traditional general medical practices in two English locations. We have termed these types of organisations ‘Alternative Providers of Primary Care’ (APPC).

Following an outline of our methods, we report the findings of the study. We conclude with a brief discussion of the significance of our findings in the context of developing policy [7,8].

## Methods

This research had two main objectives: first to understand how PCTs commission primary care, including: the process of commissioning; the specification of care to be provided and the performance management of subsequent contracts. Secondly, to understand how APPCs are organised and operated for the provision of primary medical care to the NHS, including: marketing and the impact of competition; internal organisation, including skill-mix, the organisation of work and the specification of tasks; external relationships, including identifiable impacts on surrounding practices and interactions with local professional groups such as the Local Medical Committee and local commissioning groups.

In order to address these issues we undertook two parallel case studies over a period of approximately 14 months ending in December 2010 [9]. Each case comprised a geographically-defined cluster of a single PCT (or multiple PCTs sharing commissioning functions) and some or all of its associated APPCs. The sites were purposively selected to provide a range of APPCs (including ownership types, date of procurement and size) and locations within two discrete Strategic Health Authority (geographical) areas. Data collection methods were wholly qualitative and placed considerable emphasis on observation of meetings (such as internal APPC meetings and those held between APPCs and their associated PCT) in addition to one-to-one interviews and collection of available documentation. Observations were recorded by the observer in contemporaneous field notes, and interviews were audio-recorded and transcribed with permission. In total we observed 27 meetings and conducted 23 formal interviews (each lasting about an hour) with individuals from PCTs and APPCs (management, GPs and practice managers) who were involved with the procurement, operation of and monitoring of contracts. Interviewees were identified via job titles (e.g. commissioning manager, GP manager), from observations of roles and behaviour in meetings and following recommendations from other interviewees. This allowed us to gather as wide a range of views as possible. We also undertook informal discussions with PCT representatives at the beginning of the research to gain contextual detail and an understanding of the ‘story so far’. These are reported as meetings between the researcher and PCT staff. In addition we collected documents such as tender documents and business plans (for each APPC), discussion documents, performance reports and meeting minutes relating to APPCs. These were used to provide background and contextual information about the procurement process, contract monitoring and APPC priorities and performance. A summary of the data collected can be found in Table 1.

**Table 1:** Fieldwork [9]

	Interviews	Observations
**Site 1**	16	22
**Site 2**	7	5
**Total**	23	27

In Site 1 we observed 11 pre-meetings (average 2.5hrs), 9 Joint service reviews (average 2.5 hours), and 2 others (average 2.5hrs). These included more informal discussions pre and post meetings with APPC and PCT representatives resulting in approx 55 hours of observation. In Site 2 we observed 5 meetings (average 2 hrs) resulting in approx 10 hours of observation. The imbalance in data collection is due to the differences in organisation between the two sites. In Site 1, many meetings were held (including, for example, ‘pre-meetings’ held before the formal joint service review meetings), and a significant number of PCT and other staff were involved. In Site 2, far fewer meetings were held, and the process was managed by a smaller team of people. In spite of this, the overall findings were similar between the sites, As a result of these differences, more illustrative quotes are provided from Site 1.

Site 1 was based on a PCT which was not involved in the 2006 PCT reorganisations. There were over 50 GP practices and over the previous few years several new practices have been commissioned. The structure of the PCT meant that commissioning/procurement and monitoring of primary care services were located within different Directorates, with commissioning managed by the Commissioning Directorate and monitoring managed by the Primary Care Directorate. Roles within this structure were changed during late 2009 (just prior to fieldwork commencement) which resulted in different ways of working and the development of different relationships between the people involved at both the PCT and APPCs. Following the publication of the newly elected Coalition Government’s 2010 policy intentions[7], roles again were changing due to the financial savings required to be made by PCTs. This included the announcement of redundancies and consequent role changes.

Site 2 was reorganised about two years prior to the research into a cluster of four PCTs with four statutory boards. It had a single chief executive, a single executive management team, one clinical executive and one management executive. There was a procurement and contract management Directorate, and performance management sat within the primary care and community team within this Directorate. After the 2010 election [7], the PCT was re-organised again, with the creation of a regional team for primary care commissioning, covering both procurement and contract management.

Our approach to data analysis was as follows. Primary data (field notes, transcripts and documents) were entered into Atlas.ti software, in order to both organise the large amounts of data collected and provide a space within which the team were able to work together on the analysis. First level coding [10] provided an initial categorisation of responses and incidents according to a framework developed from our research questions (see above). Later analysis included more inductive coding, enabling us to address issues not anticipated in initial research questions but which may nevertheless have important policy implications. Emerging themes and theoretical ideas were discussed and refined throughout at team meetings and through written memos, allowing precise definitions and use of codes to evolve. The sub-headings used throughout the following section represent the main themes drawn from the data collected across the two sites.

## Results and discussion

In what follows, verbatim quotes from interviews and extracts from fieldnotes and collected documentation are included where they illustrate the point being made or represent a typical response. Respondents and sites are anonymised, using broad job titles and ID numbers to preserve confidentiality.

### Procurement

The process of procurement was described as complex and centrally directed. A senior PCT officer described this:

*My understanding is that they were something that's been pushed from the centre…. I do feel that the way of...the principle of having the extended hours and the additional services*, *and the demanding targets – that all sits quite comfortably with me. The bit that I struggle with*, *is about the complicated way in which the contracts have been designed*. (Site 1, Interview PCT Officer, ID 1.2).

Procurement was consistently described as time-consuming, with very tight timetables. The work included public consultation, preparation of invitations to tender, receiving and scoring bids, moderation and dealing with legal issues. In addition, negotiation proved more difficult than expected:

*All APMS contracts to date proved difficult to negotiate - not simple like Government said would be [i.e. could be tweaked to fit local circumstances and have needed lots of legal input each time]. There was lots of interest for the 1st round* (*2008*) *fairness round BUT much less for equitable access round - providers had whole country to look at by then.* (Site 1, notes from meeting between researcher and PCT representatives, March 2010).

Processes could be contentious in terms of confidentiality and transparency and as a result the process became highly legalistic:

*I think*, *for reasons that I don't understand*, *there was a lot of tightness around the process to do with confidentiality and the fact that the...because these are not typical contracts*, *and because the costings are quite different than [for other forms of GP contract]*, *I think there's a lot of tightness around the whole process.* (Site 1 PCT Officer, ID 1.2).

Levels of interest from APPCs varied, with less interest shown by the bigger national companies (eg Virgin, United Health) in the EAPMC round than in the earlier FPCP round. This is probably explained by the fact that procurement under FPCP only occurred in a small number of PCTs, whereas every PCT was required to procure at least one new practice under EAPMC.

Core provisions of the APMS contract were set centrally and were very similar to other forms of primary care contracts; however PCTs retained some ability to address local needs. A small number of respondents commented that the larger private companies were easier to deal with as they were ‘more business-like’, but it was also said that some larger providers often failed to make their bid sufficiently locally focused. The process of procurement was new to both the PCTs involved and the potential providers themselves, and was described as a steep learning curve.

Many respondents across the two sites stressed the *cost* of procurement. Under FCPC there was central funding for the procurement process, but no extra funding for the practices procured. Under EAPMC, by contrast, recurrent money was made available to fund practices in the longer term, but no funding provided for procurement. Some central procurement advice and support was provided, but centrally imposed timescales were so tight that by the time advice or support was available, the relevant decisions had been made:

*I think the other problem for us as commissioners was getting advice from the Department of Health; that was another big problem for us*, *because we hadn’t done it before. So we were looking to the DH for advice. So things for instance*, *the scoring methodology [for assessing tenders]*, *it was very*, *very complex*, *far more complex than really it needed to be. But we didn’t know any different. So getting some clarification around some of the detail around that*, *we didn’t always get it on time*, *or the tender workshops that DH ran*, *well*, *fine*, *we’d do them*, *but they’d do them after our tender was submitted.*(Site 2, interview PCT Officer, ID 2.1)

In addition, availability of suitable premises was an issue, with several new practices initially occupying temporary accommodation. This caused problems for those affected, as they struggled to provide the full range of services or advertise their location.

### Types of APPC awarded contracts

The three main types of primary care provider organisation are GP-led companies, corporate providers and social enterprises [11] [p9]. We found a diverse mix of these types of APPC in both our Sites. Site 1 had several different APPCs, each of which held between one and five separate APMS contracts under either or both FPCP and EAPMC. In Site 2 there were over five different providers holding APMS contracts, most procured under the EAPMC round. They included private companies (sometimes in partnership with local GPs), an established out-of-hours service provider and a group aiming for vertical integration with a Foundation Trust.

It was recognised early in the process in both sites that the background and experience of individuals working within the APPCs had an impact on the operation of the contract. This PCT officer explained it thus:

*It’s evident that some practices are dealing with things better than others but I think all of them incur like problems*, *but I mean I suppose it’s like anybody the more experience you have at something… And you have different people and your different partners*, *you’ve got [APPC1] they were pharmacy*, *you know*, *pharmaceutical and then they’ve got other business managers …so some areas they will have better understanding. Then you’ve got like with [named GP APPC3]*, *s/he’s been in the GP for some time and then I think a lot of it comes down to business as well*, *so I think the role sort of competency*, *they have all got a great knowledge and some come across in different ways.* (Site 1, interview PCT Officer, ID 1.4)

Another PCT officer explained perceived differences between two of the providers in Site 1:

*I do think*, *[APPC1] are much more commercial*, *and actually that's been very refreshing because… they know what the target is*, *they know what the contract is*, *and they're working to that. So they're very clear…Whereas perhaps where we've got contractors who have had a GMS or PMS contract before and have no experience of APMS I think they have struggled to acknowledge*, *recognise that it's a very different contract*…(Site 1, interview PCT Officer, ID 1.7).

### Provider behaviour

New providers were required to provide core primary care services such as those currently provided by traditional general practices. In common with traditional practices, APPCs can also be expected to provide: ‘directed enhanced services’ (services that PCTs must provide); ‘locally enhanced’ services (which are locally required and agreed); and ‘national enhanced services’ (designed to meet local needs but commissioned to national specifications and prices) [12]. However, APPCs were also subject to additional specifications. These included the provision of longer opening times than other local GPs: at least 5 additional hours per week or a minimum of 57.5 hours, but often including stipulated opening hours of 8am – 8pm (Monday to Friday) and Saturday mornings [13]. Additionally, ‘GP-led Health Centres’ were expected to open for a minimum of 84 hours per week (8am-8pm, 7 days a week, 365 days/year), and were also required to provide facilities for ‘walk-in’ patients [14] that is, patients not registered with the practice.

Attracting new patients to the APMS practices involved leafleting, stalls in supermarkets and themed events. This often attracted criticism from local GPs. A practice manager set out some of their marketing strategies:

*We put notices up in local shops*, *clinics*, *chemists*, *there was articles in a newspaper… just before we opened and we’ve also had*, *up to now*, *two leaflet drops… within a three mile radius of the practice. And we also have an open day this coming Saturday.* (Site 1, APPC1, Interview Practice Manager ID 1.17).

As time went on, however, patient recruitment was felt to come more from personal recommendations than from marketing events. The majority of the new practices studied had struggled to meet their target list sizes, even in areas previously identified as ‘under-doctored’, so that some of the contracts were running at a loss overall. In contrast, practices providing walk-in services tended to be over-performing on this aspect of the contract.

We found few systematic differences between the ways of working adopted by APPCs and what might be expected in traditional general medical practice. Although most APPCs relied mainly on salaried GPs, the working roles and practices of clinicians within the practices did not appear to differ systematically from traditional practices. For example, APPC staff took on roles such as medical lead or Quality and Outcomes Framework (QOF which is a voluntary reward and incentive programme for all GP surgeries in England) lead, and APPC practices used standard protocols just as many traditional practices do. A number of APPCs in Site 2 had struggled to recruit permanent medical staff, and were employing extensive locum cover. In Site 1, by contrast, there was a financial penalty associated with using locum doctors, as the PCT regarded this as harmful to continuity of care. In general however, the skill-mix profile of APPC practices did not look obviously different to a traditional GMS practice, except where the former had walk-in facilities.

### Contract monitoring

In the English NHS, where quasi-markets (separation between commissioners and providers) exist, contracts have several functions: defining a level of activity required for payment to be achieved; setting out a specific timescale for these goals; detailing quality and standards; and setting out penalties should these not be met [15]. This enables monitoring to be undertaken and is generally agreed between the commissioner and provider as part of the procurement process. Historically, general practice contracts have been fluid, flexible and largely vague [13] (often referred to as the ‘John Wayne contract’: ‘a GP’s gotta do what a GP’s gotta do’). However, the monitoring of the APMS contracts was much tighter and more formal than for other primary care contracts, as explained by a PCT Officer:

*it’s more formal*, *obviously*, *it’s…you can build up relationships with GMS and PMS and advise on what best practices and steer them in the right direction but with APMS you have the tools there to ensure that they’re delivering in the areas that are set out in the KPI [Key Performance Indicators].* (Site 2, interview PCT Officer, ID 2.4)

Monitoring included submission of statistics and electronic workbooks, sampling and checking (e.g. practice appointment availability), and face-to-face meetings between PCTs and contractors. All of these methods fed into an assessment of performance against an extensive range of Key Performance Indicators (KPIs), and payment was linked to achievement. In Site 1 this process was very formal with several meetings each month between the PCT and each provider at which providers were expected to answer detailed questions. In Site 2, whilst KPIs were similar, monitoring was less intensive, and was more likely to involve one to one meetings between a practice lead and a PCT manager.

Achievement of KPIs in both Sites was worth 25 percent of the total contract value. KPIs were organised into the following areas: access; quality; service delivery; value for money; and patient experience. These domains were centrally set with detailed examples provided by the Department of Health in a contract template. Within this, however, the PCTs could specify the required KPIs (for our sites each domain contained up to 21 specific indicators) and weight them according to local priorities.

During the research in both sites we saw the negotiation of formal contract amendments, as it became clear that some KPIs were unclear or unworkable. The formality of this process and the associated managerial time required is in contrast to the informal flexibility associated with a traditional GMS contract. Examples included discussion in Site 2 of whether or not it was legitimate for a patient who arrived at a walk-in centre 10 minutes before closing time to be turned away. In Site 1 the ongoing need for renegotiation was further complicated by the fact that a different team was involved in monitoring the contract from that which undertook the procurement. This meant that the staff responsible for monitoring the contract did not know what the thinking behind the contract provisions had been, and did not understand some of the KPIs. In Site 2 these teams overlapped, ensuring that those monitoring the contract were aware of the intentions behind individual clauses.

There was some evidence that the experience of monitoring APMS contracts in this way led PCT staff to think about traditional practices in a different way and in some cases consider introducing new targets into existing contracts:

*The targets and KPIs in the APMS contracts are very specific to the APMS contracts. We are trying to introduce*, *as part of our health and equality*, *some stretch targets for our GMS and PMS contracts*, *but that’s something that's just currently in process that we’re trying to get worked out and agreement with the LMC...* (Site 1, interview PCT Officer, ID 1.2).

Nevertheless, it was recognised that the monitoring process used was very intensive and time consuming.

### Externalities

PCT respondents in Site 2 felt that the procurements had ‘stirred up’ local GPs and made them think a bit more about their own services, making them ‘raise their game’ in relation to other local practices. For example, the PCT described the procurements as ‘giving them leverage’ over GPs, and it has also created a market in alternative providers. This was obvious when they went out to tender for the local contract for Out of Hours Services: after the EAPMC process there were a number of new providers in the market. In addition, we were told that the process had prompted some GPs locally to extend their opening hours [9].

One of the managers from APPC1 in Site 1 explained their belief that tension could be healthy:

*Take PBC [Practice-based Commissioning] – in some areas PBC groups tried to stop APPC1 coming in. It is stacked against us from [the] start e.g. PEC Chairs* (*who are GPs and have friends locally*) *chairing commissioning panels. However I would say you need a healthy tension in an area – this can push some practices under but those that can take the lead and make things better.* (Site 1, APPC1, Interview APPC Manager, ID 1.14).

There had been some difficulties in the relationships (at least initially) between APPCs and other local GP practices, especially where they were based in the same building. In Site 1 there were allegations by APPCs that other practices had removed signage and misdirected patients. In Site 2 there were suggestions that staff at a minor injuries service which shared premises with an APPC practice had deliberately misdirected patients away from the APPC. These difficulties seem to stem from competition over patients:

*Obviously GPs were established prior to this scheme. There were a number of anxieties*, *as there were nationally*, *especially around the health centres*, *would they take lots of activity away from their practice*, *the fact that they were open longer hours and at weekends.* (Site 2, interview PCT Officer, ID 2.3)

APPC practices were contractually required to participate in PBC; however some did not. This was sometimes out of choice but often due to reluctance of existing PBC groups to accommodate the new practices into their structure

*We’re not part of PBC. That’s quite interesting actually because contractually we have to be part of PBC and so we started with the intent of*, *you know*, *being part of the PBC because from a contractual point of view*, *you know*, *we’re required to do that. The PBC group then*, *supported by the PCT*, *said no you’re not going to*, *we don’t want you here so don’t come please. And then the PCT said well if that’s what they want then that’s fine with us.*(Site 2, APPC 4, Interview Executive Director, ID 2.6)

Additionally, for those APPC practices which were engaged with PBC, the absence of full time GPs could make this difficult and sporadic.

### Perceived difficulties and successes

All interviewees were asked about their perceptions of the process as a whole. Problems reported included difficulties in attaining predicted list sizes and employing full-time GPs; turnover of GPs; co-location of practices with established practices; and definitions or interpretations of KPIs within the contract. Other commonly identified issues included the affordability of the APPC contracts with small lists, as many of the indicators were based on a projection of increased list size over the life of the contract. The following was observed at an end of year reconciliation meeting:

*There followed a brief discussion between APPC1 and PCT1 about the fact that all APMS practices had been given the same list growth projections at the outset and the APMS practices had not been able to change this. It did not make sense as in addition to this they were provided with practice demographics and these were highly variable so both agreed at outset they should have been able to change the projections* (*set by centre*)*.* (Site 1, notes observation, APPC1 reconciliation meeting September 2010)

There was also some concern that the existence of ‘walk-in’ centres was stimulating new demand for services which could significantly add to overall costs (over performance against contracts). Additionally, both PCT and APPC staff again highlighted the issue of time taken in monitoring the contract.

All respondents were asked what their definition of ‘success’ for an APMS contract would be. The answers varied but often included measures such as meeting KPIs and QOF targets; achieving patient satisfaction rates; increasing list size and financial stability, renewal of contracts after the initial 5 years; staff stability, high morale and a well functioning practice; provision of services under adverse circumstances (e.g. poor premises); provision of additional services and good working relationships.

Most commonly, an answer included meeting KPIs and QOF targets plus some sort of broader measure such as references to providing better patient care or improving health as illustrated by the following answers:

*My definition of its success is improving the health of the local community*, *our registered patients*, *yes*, *but the local community as a whole. So I think that’s a difficult one because that’s just an aspiration we have as an organisation and not specifically related to this sort of APMS contract.* (Site 1, APPC3, interview Business Manager ID1.25).

*Well obviously taking into consideration the KPIs and it's a big thing for us*, *and QOF*, *it's the idea behind them is...the reason that they're there is to provide better patient care and to check that we are doing that. I suppose it's like examination results for schools. It's just a way of checking we're doing what we should be doing*, *and that's the overall thing that just because something's a KPI we don't focus on it*, *we focus on the full patient care as well.* (Site 1, APPC1, interview Practice Manager, ID 1.16)

Patient satisfaction rates were measured within the APMS contracts and as part of QOF and were worth a substantial amount of money. They were thus perceived to be important by both PCT and APPC respondents:

*Well*, *from a wide perspective*, *a successful contractor is one that achieves its KPI’s and hits its list size targets*, *because then it’s doing what was expected of it from the outset. From a personal point of view*, *I would say …the KPI’s were set for a reason*, *they were the KPI’s that the PCT wanted the practice to achieve in. So if they are hitting those KPI’s then that would be a successful practice in my eyes. Obviously*, *to ensure that there’s no complaints from patients*, *we have a PALS service and the providers are also required to let us know of any complaints that they have received. So*, *obviously*, *patient satisfaction would be a good factor in deciding if it’s successful or not.* (Site 2, interview PCT Officer, ID 2.4)

Only a few PCT staff and APPC business managers suggested that the new arrangements should be judged in terms of the provision of greater access and choice that had been the official rationale for the whole centrally-directed procurement programme.

We asked all of our respondents if they thought that the APMS contracts provided value for money. Many were unsure, as they suggested it was taking time for the contracts to bed in. This PCT officer put it thus:

*It depends*, *it depends why they’re there. If they’re to stir up the GP community to*, *then yeah*, *they’re a good thing. If*, *if*, *as a strict economic model*, *probably not.* (Site 1, interview PCT Officer, ID 1.6)

Others thought they were definitely not value for money:

*I absolutely don't think its value for money*, *when you think about what a GP gets*, *it's sixty five quid a year per patient*, *and a walk in centre gets more than that per walk in...Every time someone walks through the door*, *no*, *I don't think it's value for money*, *absolutely not.... It's very costly*, *it's not value for money*, *it just isn't value for money.* (Site 2, interview PCT Officer, ID 2.2)

This senior PCT Officer added that GP-led health centres are massively over-performing on their walk in contracts and were thus ‘not sustainable’.

## Discussion

The commissioning of primary care did not form a prominent part of the 2010 Health White Paper, *Equity and Excellence*[7], other than to state that it would be become the responsibility of the new NHS Commissioning Board (NHSCB). There was no specific reference to the different types of primary care contract, but subsequent guidance has suggested that from April 2013 the responsibility for GP-led health centres established under EAPMC and other APMS contracts will be taken on by the NHSCB [17]. This guidance suggests that Local Area Teams of the NHSCB will take over responsibility for monitoring APMS contracts, although it seems unlikely that they will be able to maintain the level of detailed performance management that we observed in our study, as they will have significantly fewer staff than PCTs locally.

Emerging Clinical Commissioning Groups (CCGs – the organisations which will replace Primary Care Trusts in commissioning the majority of health services locally from April 2013 and formally known as GP consortia) should not inherit any contractual liabilities for the services provided under current EAPMC or APMS contracts [18], although they may take on responsibility for administering some aspects of both GMS and APMS contracts (for example, QOF and improving the quality of primary medical care) [17]. It nevertheless seems clear that future policy will be based on the assumption that competition between providers is essential in any drive to improve quality [7] [p37].

It is therefore implied that service provision by APPCs will continue, although it is unclear whether the full range of currently available primary care contracts (APMS, PMS, GMS) will continue to be available over time, or if there will be a convergence between the types, and whether the five-year duration of contacts with retendering (common under APMS) could be extended to other types. Whilst our study is a small one, our evidence is detailed and robust. There is therefore the potential to learn from our research about the future procurement, operation and monitoring of such providers working under the APMS regime, and for future procurement by the NHS Commissioning Board.

Other research [19] has drawn a distinction between relational and (classical) transactional contracting. In the former, existing relationships and history of working can shape the way in which commissioning / contracting is undertaken with less formal processes, flexible timetables and little use of formal penalties. Transactional commissioning / contracting is much less fluid, sticking more rigidly to specified timescales and targets (e.g. KPIs) and having the ability to penalise poor performance and ultimately terminate contracts.

It was clear from this research that the contracts (and processes) we observed were of a transactional nature. This is, perhaps, inevitable, given that fact that these contracts were with new (and often untried) providers, with whom PCTs did not have any prior relationships. However, this study shows that, whilst transactional contracting may be safe, in that it ensures that providers do provide the services they are contracted to provide, it is also hugely time consuming and expensive, and perhaps therefore unsustainable in the new NHS [20].

Furthermore, it is also probably less effective overall, as demonstrated by the detailed discussions that we observed over how to interpret the detailed KPIs. Thus, for example, the discussion observed between APPC and PCT managers around whether or not a patient who presented 10 minutes before official ‘closing time’ could legitimately be turned away; under the more flexible and less clearly specified GMS contract it is unlikely that this would be seen as an option. The only significant advantage that these contracts would seem to have is that they are easier to terminate if they are not felt to be operating effectively.

It appears that concerns such as those we have identified from our studies may already have caused closure or temporary suspension of some GP-led health centres (commissioned under EAPMC). For example, one closure [21] in Stockport in September 2010 and in November 2011 the closure of a walk-in centre in central Leeds as part of moves to save money [22]. From 1 December 2011 it was reported that the Hillside Bridge walk-in centre, Bradford (the first Darzi centre to be opened in December 2008), would be open from 2pm to 8pm, seven days a week – a reduction in its original opening hours which was 8am to 8pm. Any patients registered there can still access appointments from 8am; the changes only affect non-registered patients using the walk-in service [23]. These amendments / closures are made possible by the transactional nature of the contracts involved, and this could be seen as an advantage of this approach. However, such changes are experienced as disruptive by patients [24], and it seems unlikely that a model of primary care provision predicated upon the relatively rapid entry and exit of new providers into a market would be seen generally as desirable in the context of an NHS in which building a relationship with a known and trusted GP is regarded as important by both doctors and patients [25].

We could not formally assess outcomes over the short duration of this project, but respondents did discuss their own perceptions of success. Some told us that thinking about contracts in this way had encouraged them to be a little more challenging with existing GPs, and it may be that in the longer term this will be one of the more lasting outcomes from the process. Additionally, some claimed that the existence of local competition had led some traditional general practices to ‘raise their game’. Whilst many new providers were proud of what they had achieved, and demonstrated commitment to patient care and satisfaction, the APMS contracts whose operation we observed were generally perceived as a relatively expensive way of providing primary care, particularly in view of the difficulties many APPCs found in building up adequate list sizes. This latter suggests that the demand for new GP practices was less than policy makers anticipated, whilst the high levels of ‘walk in’ access were regarded by our PCT respondents as representing an unsustainable lowering of the threshold for seeking help and a consequent increase in costs, rather than an uncovering of important levels of unmet need.

It remains to be seen in the longer term whether APMS contracts such as these become a blueprint for the wider renegotiation of the current GMS contract, or whether they continue at the margins, ‘stirring up’ existing practices to raise their game but without wider impact. Our research suggests the need for a debate about the operation of a market in primary care provision in the longer term, set against the background of current organisational changes (development of CCGs, NHSCB) including the trade-offs between transparent processes, fair procurement, performance assurance and costs.

## List of abbreviations used

APMS: Alternative Provider Medical Services; APPC: Alternative Providers of Primary Care; EAPMC: Equitable Access to Primary Medical Care; FCPC: Fairness in Primary Care Procurement; GMS: General Medical Services; KPI: Key Performance Indicator; NHS: National Health Service; NHS NSR: NHS Next Stage Review; NHSCB: NHS Commissioning Board; PALS: Patient Liaison Service; PCT: Primary Care Trust; PMS: Personal Medical Services; QOF: Quality and Outcomes Framework.

## Competing interests

There are no financial or other competing interests for any of the authors involved in the research or writing this paper.

Ethical approval for the study was given by North West 9 Research Ethics Committee, and research governance approval obtained from the relevant PCTs.

## Authors’ contributions

All authors contributed to the conception and design of this research. Almost all the fieldwork was undertaken by AC and IM. Data collected was discussed at team meetings which included all four authors and first level coding (organising documents, interviews and observations into themes) was undertaken by AC and IM using Alas Ti., as was drafting the original manuscript. All four authors were involved in critically revising and editing it and all have read and approved the final manuscript.

## Information about authors

All authors currently work within the Health Policy, Politics and Organisation Group (HiPPO), within the Institute of Population Health at Manchester University. HiPPO and the Service Delivery and Organisation research group at the London School of Hygiene & Tropical Medicine (LSHTM) constitutes the Department of Health Policy Research Unit in Commissioning and the Healthcare System (PRUComm). Our analytical work supports understanding of how commissioning operates and how it can improve services and access, increase effectiveness and respond better to patient needs. It should be noted that this work was undertaken when HiPPO was part of the National Primary Care Research and Development Centre (NPCRDC), Manchester University.

## Declarations

Publication of this supplement was funded by the Nuffield Trust.

This article has been published as part of *BMC Health Services Research* Volume 13 Supplement 1, 2013: The Limits of Market-based Reforms. The full contents of the supplement are available online at http://www.biomedcentral.com/bmchealthservres/supplements/13/S1.
